# Two‐photon microscopic observation of cell‐production dynamics in the developing mammalian neocortex in utero

**DOI:** 10.1111/dgd.12648

**Published:** 2020-01-14

**Authors:** Ryotaro Kawasoe, Tomoyasu Shinoda, Yuki Hattori, Mami Nakagawa, Trung Quang Pham, Yoshihiro Tanaka, Ken Sagou, Kanako Saito, Satoru Katsuki, Tomomi Kotani, Akihito Sano, Toshihiko Fujimori, Takaki Miyata

**Affiliations:** ^1^ Anatomy and Cell Biology Nagoya University Graduate School of Medicine Nagoya Japan; ^2^ Division of Embryology National Institute for Basic Biology (NIBB) Okazaki Japan; ^3^ Robotics Lab Department of Electrical and Mechanical Engineering Graduate School of Engineering Nagoya Institute of Technology Nagoya Japan; ^4^ Department of Obstetrics and Gynecology Nagoya University Graduate School of Medicine Nagoya Japan

**Keywords:** cell division, cerebral cortex, in utero, neural progenitor cells, two‐photon microscope

## Abstract

Morphogenesis and organ development should be understood based on a thorough description of cellular dynamics. Recent studies have explored the dynamic behaviors of mammalian neural progenitor cells (NPCs) using slice cultures in which three‐dimensional systems conserve in vivo‐like environments to a considerable degree. However, live observation of NPCs existing truly in vivo, as has long been performed for zebrafish NPCs, has yet to be established in mammals. Here, we performed intravital two‐photon microscopic observation of NPCs in the developing cerebral cortex of H2B‐EGFP or Fucci transgenic mice in utero. Fetuses in the uterine sac were immobilized using several devices and were observed through a window made in the uterine wall and the amniotic membrane while monitoring blood circulation. Clear visibility was obtained to the level of 300 μm from the scalp surface of the fetus, which enabled us to quantitatively assess NPC behaviors, such as division and interkinetic nuclear migration, within a neuroepithelial structure called the ventricular zone at embryonic day (E) 13 and E14. In fetuses undergoing healthy monitoring in utero for 60 min, the frequency of mitoses observed at the apical surface was similar to those observed in slice cultures and in freshly fixed in vivo specimens. Although the rate and duration of successful in utero observations are still limited (33% for ≥10 min and 14% for 60 min), further improvements based on this study will facilitate future understanding of how organogenetic cellular behaviors occur or are pathologically influenced by the systemic maternal condition and/or maternal‐fetal relationships.

## Graphical abstract

Intravital two‐photon microscopic observation of division of neural progenitor cells in the developing cerebral cortex was performed in utero.

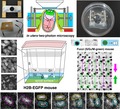

## 1 INTRODUCTION

The production of new cells by division of progenitor cells is fundamental to morphogenesis and organ development. In three‐dimensional environments, cell production via mitosis depends not only on the progression of the cell cycle but also on the movement of cells’ nuclei/somata into place for mitosis, making the overall cell‐production events highly temporally and spatially dynamic (Keller, Schmidt, Wittbrodt, & Stelzer., 2008; Kurotaki, Hatta, Nakao, Nabeshima, & Fujimori, 2007; Miyata, 2008; Norden, Young, Link, & Harris, 2009; Taverna & Huttner, 2010). Recent studies using slice culture‐based imaging and mechanical assessment of developing mammalian brain walls have shown that such dynamic cytogenetic events occur at a very high density under physiologically crowded cellular conditions (Miyata, Okamoto, Shinoda, & Kawaguchi, 2015; Nagasaka et al., 2016; Okamoto et al., 2013; Okamoto, Shinoda, Kawaue, Nagasaka, & Miyata, 2014; Saito, Kawasoe, Sasaki, Kawaguchi, & Miyata, 2018; Shinoda et al., 2018); the studies also show that delays in the cell cycle or nuclear migration in a subpopulation of progenitors can easily lead, in a cell non‐autonomous manner, to secondary (more widespread) disorganization of histogenesis (Okamoto et al., 2013; Watanabe, Kawaue, & Miyata, 2018).

**Figure 1:**
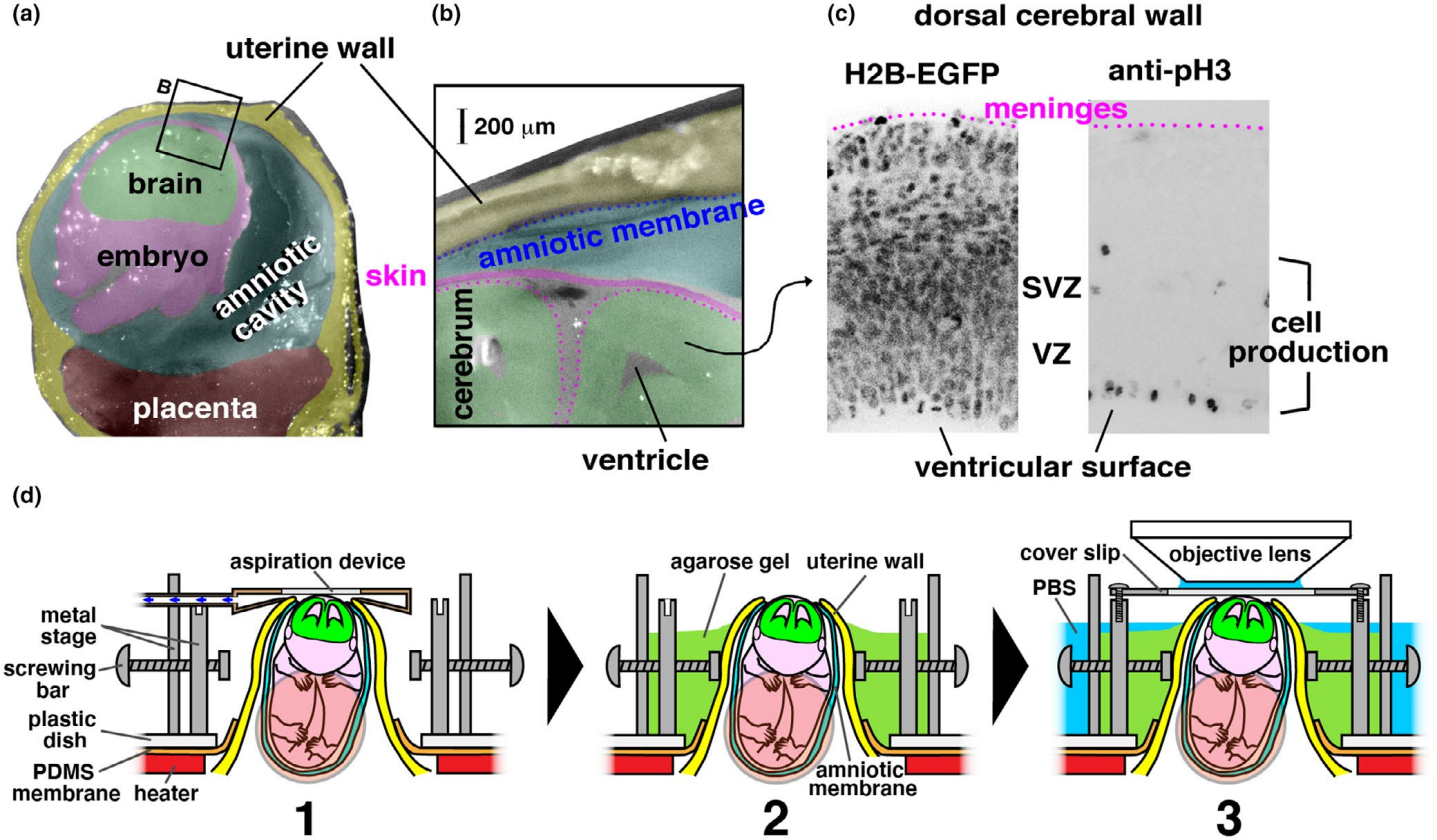
Objective and methods of in utero two‐photon microscopic live observation. (a) Cross‐section photomicrograph of the uterus and the amniotic cavity containing an E14 mouse embryo (cut was coronal at the head of the embryo and through the placenta). (b) Magnified view of the uterine wall, the amniotic membrane, the amniotic cavity, and the dorsal portion of the embryo's head. (c) Photomicrographs of the dorsal cerebral wall where either the H2B‐EGFP signal (all cell nuclei and chromosomal condensation during M phase) was observed live or M‐phase cells were visualized immunohistochemically with anti‐phosphohistone H3 (pH3). VZ, ventricular zone. SVZ, subventricular zone. (d) Steps (1–3) and devices for posturing and holding in utero growing embryos are shown

Given the emerging importance of cell‐production dynamics under physiologically crowded conditions where numerous mammalian cells congregate, it is necessary to ask how dynamic cell‐production events exist in in vivo situations. Recent studies successfully combined exo utero whole‐embryo culture of mice and light‐sheet microscopy to monitor cell‐production dynamics from embryonic day (E) 5.5 to 8.5, extensively capturing cell divisions and cell‐cycle‐associated nuclear migration (Ichikawa et al., 2013, 2014; McDole et al., 2018). In embryos/fetuses growing in utero at more advanced stages, however, cell‐production dynamics have not yet been directly monitored. Nevertheless, following the pioneering electrophysiological studies carried out on in vivo fetuses (or those partly removed from the uterus but maintaining connection with the mother via placental blood flow; Fitzgerald, 1987; Sakaguchi & Nakamura, 1987), intravital two‐photon microscopy (2PM) of fetuses was recently performed to observe tangential migration (Ang, Haydar, Gluncic, & Rakic, 2003; Higuchi, Kita, & Murakami, 2016; Yanagida, Miyoshi, Toyokuni, Zhu, & Murakami, 2012) and to observe calcium oscillations (Yuryev et al., 2016) of young neurons in the mid‐ or late‐embryonic cerebral cortex. These in vivo imaging studies on developing mammalian brains motivated us to establish a method for in utero 2PM imaging of division and mitosis‐associated interkinetic nuclear migration (IKNM) of neural progenitor cells (NPCs) in the cerebral cortex at E13–E14 (Figure 1a–c) using H2B‐EGFP (Kurotaki et al., 2007) or Fucci (Abe et al., 2013; Sakaue‐Sawano et al., 2008) transgenic mice.

## 2 MATERIALS AND METHODS

### 2.1 Animals

Animal experiments were conducted according to the Japanese Act on Welfare and Management of Animals, Guidelines for Proper Conduct of Animal Experiments (published by Science Council of Japan), and the Fundamental Guidelines for Proper Conduct of Animal Experiment and Related Activities in Academic Research Institutions (published by Ministry of Education, Culture, Sports, Science and Technology, Japan). All protocols for animal experiments were approved by the Institutional Animal Care and Use Committee of Nagoya University (No. 29006) and the National Institutes for Natural Sciences (19A021). Fucci mice (Fucci S/G_2_/M‐Green#504, accession number RBRC02706; Sakaue‐Sawano et al., 2008) were provided by Atsushi Miyawaki (RIKEN, Japan). R26‐H2B‐mCherry transgenic mice (accession No. CDB0239K; Abe et al., 2011) used at Nagoya University were provided by Toshihiko Fujimori (NIBB, Japan). R26‐H2B‐EGFP transgenic mice (Kurotaki et al., 2007) were maintained and used at the NIBB. E0 was defined as the day of vaginal plug identification.

### 2.2 Slice culture

Cultures of cerebral walls were prepared as described previously (Saito et al., 2019; Shinoda et al., 2018). Briefly, cerebral walls were microsurgically processed and embedded (ventricular surface top) in a polystyrene cell culture dish (Corning) with Atelocell IAC‐30 collagen gel (Koken) at a concentration of 0.8 mg/ml. Confocal time‐lapse images (*en face*, parallel to the ventricular surface) were obtained with an upright BX51W1 microscope (Olympus) equipped with a CSU‐X1 laser scanning confocal unit, a 60 × objective lens (LUMFL N 60XW, N.A. = 1.10 Olympus) and an iXon + EMCCD camera (Andor); an on‐stage culture chamber (Tokai Hit) was filled with 45% N_2_, 40% O_2_, and 5% CO_2_ on a CV1000 system (Yokogawa) with a 60× objective lens (PlanApo 60×/1.40 Oil, N.A. = 1.40 Olympus).

### 2.3 Intravital imaging of neural progenitor cells using two‐photon microscopy

**Figure 2:**
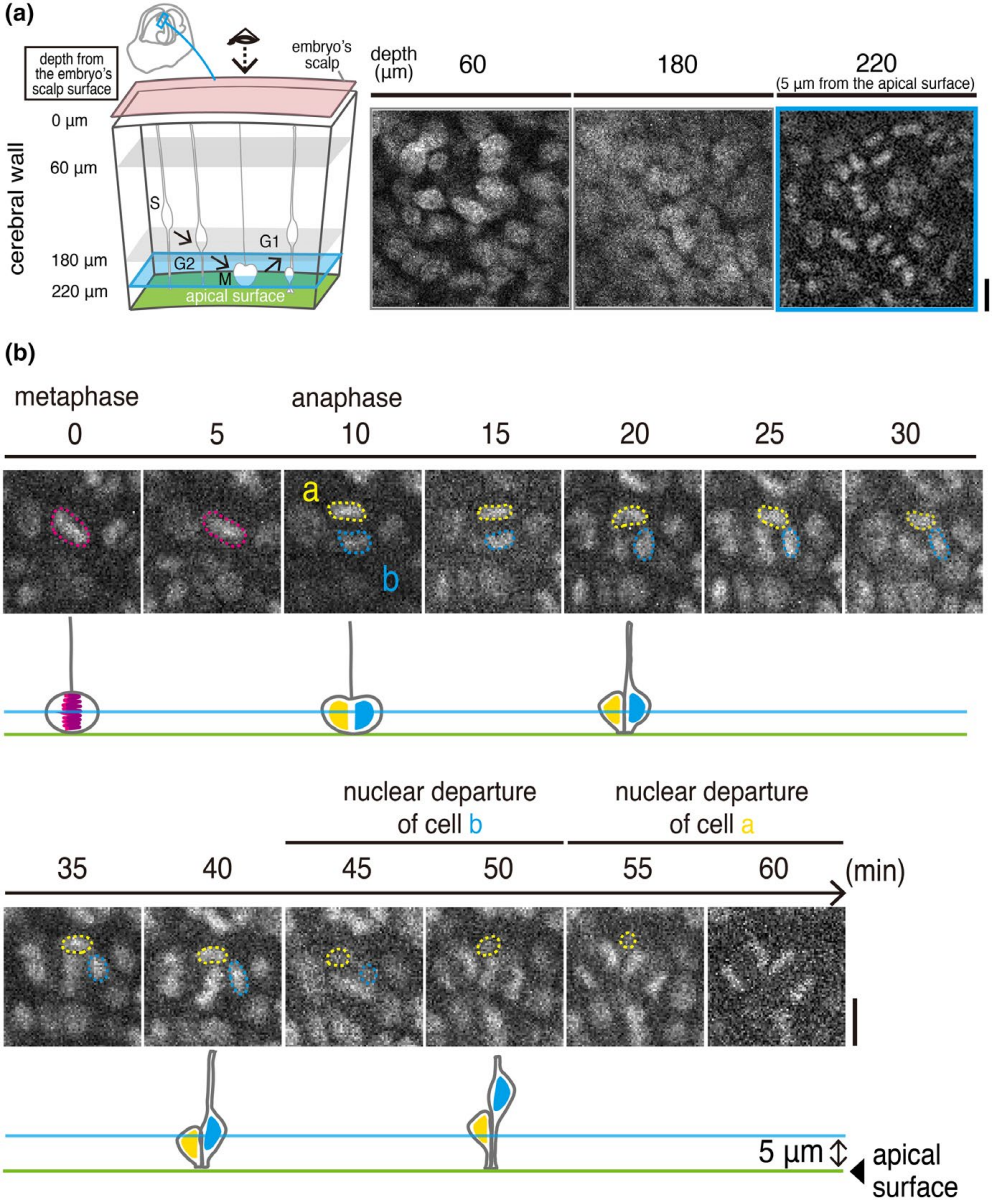
In utero 2PM view of an E13 H2B‐EGFP transgenic mouse embryo enables observation of cell‐production behaviors by NPCs near the apical surface of the cerebral wall. (a) Schematic display of our 2PM scanning procedures until reaching 5 μm from the apical surface; the accompanying obtained H2B‐EGFP images are shown (see also Movie S1). (b) An example of time‐lapse monitoring of the division of an NPC and the IKNM exhibited by its daughter cells. Magenta circle, metaphase plate (chromosomal condensation) of a mitotic mother cell. Yellow and cyan circles, daughter cells' condensed chromosomes and nuclei. See also Movie S2. Scale, 10 μm

**Figure 3:**
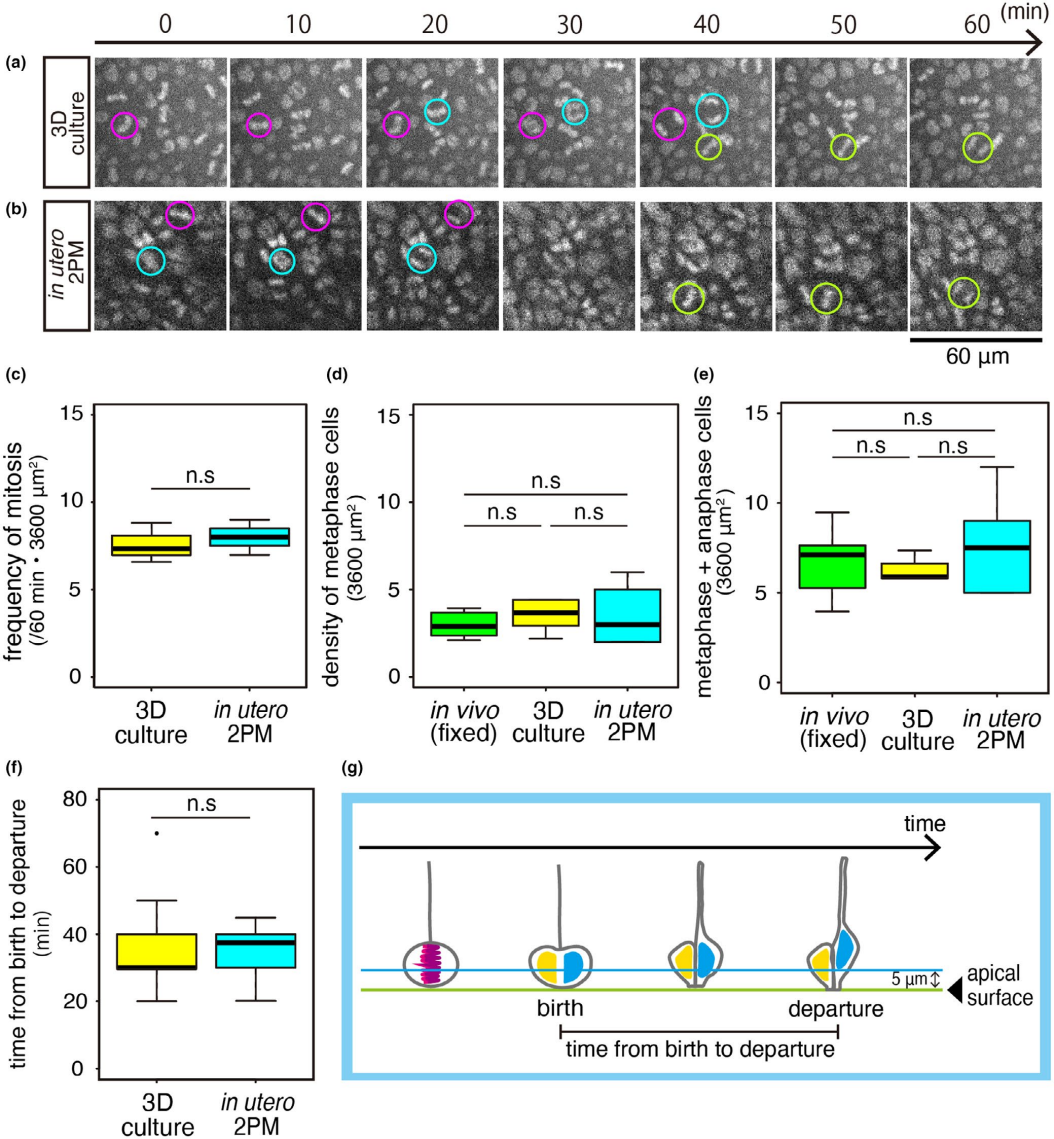
Comparison of the frequency of NPC division observed at the apical surface of E13 cerebral walls using 3D culture and in vivo 2PM. (a) Time series of an E13 cerebral wall (H2B‐mCherry in toto‐labeled dorsolateral pallium, confocal microscopically recorded 5 μm from the apical surface, 60 μm × 60 μm field). (b) Intravital 2PM time series of an E13 cerebral wall (H2B‐EGFP in toto‐labeled dorsolateral pallium, 5 μm from the apical surface, 60 μm × 60 μm field). Cyan, magenta, or green circles indicate separation of a metaphase plate into two, indicative of an NPC's division. See also Movie S3. (c) The graph shows the frequency of NPC division (judged based on separation of metaphase plates as shown in a and b) per area and period (60 min, 3,600 μm^2^) between an E13 cultured cerebral wall and the cerebral walls observed with in utero 2PM. *p* = .7 (*n* = 3, exact Wilcoxon rank ‐sum test). n.s., not significant. (d) A graph comparing the density of metaphase cells (identified in six 60 μm × 60 μm fields of snapshot pictures) is shown. (e) A graph comparing the density of cells in metaphase or anaphase (identified in six 60 μm × 60 μm fields of snapshot pictures) is shown. (f) A graph comparing the time from the beginning of anaphase until giving rise to two daughter cells (which was roughly regarded as “birth” of the daughter cells) and the “departure” (disappearance from the 5 μm level from the apical surface, illustrated in g) of the nucleus of one (earlier‐leaving) daughter cell (Shinoda et al., 2018) is shown. *p* = .5563 (exact Wilcoxon rank‐sum test; *n* = 13 in in utero 2PM, *n* = 17 in cerebral wall culture)

Pregnant mice were anesthetized by inhaling 1.5%–2.5% isoflurane (FUJIFILM) in a stream of mixed gas (55% N_2_, 45% O_2_). The body temperature of the mice was maintained at 37°C using a small‐animal warmer device (BWT‐100A, Bio Research Center), which was monitored with a rectal thermosensor (RET‐3, Physitemp Instruments). To facilitate the handling of uterine horns, 2 mg/kg ritodrine hydrochloride (FUJIFILM), a myometrium relaxant, was intraperitoneally administered. A midline laparotomy was performed, and the left or right uterine horn in the abdominal cavity was exposed. Above the exposed uterine horn, a custom‐made metal stage was set (Figure S1A, B, and E). The metal stage was covered with a pad for heating (37°C; Figure S1F,G) and on it was placed a 10‐cm‐diameter polystyrene dish whose center was apertured (2.5 cm diameter) and covered with a polydimethylsiloxane (PDMS) membrane (Figure 1d, 1). The PDMS membrane was cross‐incised (Figure S1H) to narrowly pass through a uterine horn (which was vertically pulled out from the spine‐positioned mother mouse to take a hairpin loop‐like shape). After passing the uterine horn through, a uterine sac containing the embryo to be observed was chosen, and the orientation of the embryo was manually adjusted so that the dorsolateral part of the cerebral hemisphere was closest to the objective lens. Then, a small incision was made in the uterine wall and the amniotic sac to expose the embryo's head, and cyanoacrylate glue (Konishi, Japan) was applied to the cutting edges to prevent excessive opening of the uterine window, thereby preventing accidental popping out of the embryo and detachment of the placenta from the uterus. The embryo's head was then transiently immobilized using a custom‐made plastic device for gentle aspiration (0.7–1.0 kPa; consisting of an air pump [MV‐6005V, TAIYO company] and a pressure sensor [VUS‐31R‐NV‐01, Pisco]; Figures 1d, 1 and S1I), and two screwing bars were attached to hold the uterine sac containing that embryo. After filling the space around the uterine tube and sealing the PDMS incision with agarose gel (low‐melting temperature, 3% w/v, made in phosphate‐buffered saline [PBS], pH 7.4; FUJIFILM), the aspiration device was removed (Figure 1d, 2), and the embryo's head was covered with a glass coverslip (Figure 1d, 3).

Two‐photon microscopy observations of the dorsolateral cerebral wall were performed using two systems: an A1RMP 2PM system (Nikon, Japan; in Division for Medical Research Engineering, Nagoya University Graduate School of Medicine) equipped with Mai Tai DeepSee (Spectra Physics) and a 25× objective lens (CFI75 APO 25XCW, N.A. = 1.10, Nikon) was used for Fucci (S/G_2_/M‐Green) imaging, and a TCS SP8 MP 2PM system (Leica, Germany; in Advanced Bioimaging Support, National Institute for Basic Biology) equipped with InSight DeepSee (Spectra Physics) and a 25× objective lens (HCX IRAPO L25x/0.95W, N.A. = 0.95 Leica) was used for H2B‐EGFP imaging. Imaging was performed with the following settings: scan speed, 2 fps; resolution, 512 × 512 pixels; and z‐interval, 2 μm. Excitation of EGFP (in H2B‐EGFP mice) and mAG (monomeric Azami Green) fused to geminin in Fucci mice was carried out by 900 nm laser illumination. Based on results of our pilot trials, we have set the interval of our standard intravital 2PM on NPCs in VZ of E13 or E14 mice in utero to be 5 min. Healthiness of fetuses was evaluated based on flows of erythrocytes in subdermal vessels, which were autofluorescently visualized under mercury lamp illumination, or those in cortical walls, which were visualized under 2PM excitation (Yuryev et al., 2016); fetal heart beating was used to determine the endpoint of observation. In most cases, in utero imaging was performed only for one fetus, and a second fetus, a littermate of the fetus observed first, was only rarely subjected to additional imaging through reimmobilization of a portion of the second uterine sac. In total, 21 observation trials were performed using the present protocol. In addition to these 21 cases, we experienced 40 more trials, which were all unsuccessful and consisted of trials in the early/pilot stage of this study under fluctuating (primitive) protocols and trials that had to use (after establishing the present protocol) too short uterine tubes.

## 3 RESULTS

### 3.1 Finding practical ways to optically access the places of brain‐cell production in utero

#### 3.1.1 The dorsal cerebral wall at E13–14 was chosen

Slice culture‐based assessment of cell divisions and cell‐cycle‐associated nucleokinesis (interkinetic nuclear migration, IKNM) of neural progenitor cells (NPCs) in the mouse telencephalic/cerebral wall has been extensively performed at E13 and E14 (Konno et al., 2008; Miyata, Kawaguchi, Okano, & Ogawa, 2001; Shinoda et al., 2018; Watanabe et al., 2018). Therefore, we decided to establish a method for in utero two‐photon microscopy (2PM) at these embryonic days, which should be useful for comparing 2PM results to those from slice cultures and in vivo situations. We surveyed the cell‐production zone (called the ventricular zone, VZ, approximately 100 μm thick) to find the best place for minimizing the distance between the objective lens and the VZ, and we found that the dorsal portion of the cerebral wall (future cerebral cortex) was most appropriate (Figure 1a–c).

#### 3.1.2 The embryo's scalp was kept intact

Previous in vivo embryonic (E14–E16) 2PM studies surgically removed the scalp and primitive skull‐like tissues over the cerebrum (Ang et al., 2003; Higuchi et al., 2016; Yanagida et al., 2012) to improve visibility of brain cells. However, we found that the scalp and the underlying cranial mesenchymal tissues before forming the skull at E13–E14 combined to be approximately 50 μm thick dorsally (though progressively thicker laterally/ventrally), and in vivo visibility of VZ can be achieved if we chose the dorsal region without removing the scalp and the subcutaneous tissues (which should be advantageous for studying biological issues such as developmental brain‐cranium interactions: discussed later).

#### 3.1.3 Depth of zones of interest

From the surface of the dorsal scalp at E14, the outer border of VZ (where S‐phase nuclei are abundant, which is close to the subventricular zone, SVZ) was within 300 μm anteriorly and within 250 μm posteriorly, and the end of VZ (i.e., the ventricular/apical surface where NPC mitosis occurs most frequently, Figure 1c) was within 400 μm anteriorly and within 350 μm posteriorly. At E13, the VZ was closer to the scalp surface; its apical surface was within 300 μm.

#### 3.1.4 The uterine wall and amniotic membrane were windowed

Previous intravital 2PM studies (Higuchi et al., 2016; Yanagida et al., 2012; Yuryev et al., 2016) opened the uterus and the amniotic sac, and the embryos were fully exposed. Our pilot 2PM imaging *across* the intact uterine wall (100–200 μm thick at E13–E14, Figure 1a,b) that targeted superficial neuronal zones revealed that cells within 200 μm of the embryo's scalp surface could be clearly observed (Y. Hattori et al., unpublished data); however, deeper visibility (needed for VZ) was not fully obtained in that bona fide in utero observation (i.e., optimal visibility from the objective lens through the uterine wall was within 400 μm). We therefore decided to perform the present study focusing on the VZ by opening a small window in the uterine wall (and on the amniotic membrane), as in Ang et al. (2003), while the embryo was physically kept “in utero” (in the narrowly windowed uterine sac).

#### 3.1.5 Posturing and holding of in utero growing embryos

To avoid influences of mother's respiration and vascular pulsations on microscopic observations of embryos, the uterus (pretreated with ritodrine hydrochloride to prevent contractions) was partly pulled out from the mother's abdominal cavity. The uterine tube taking a hairpin loop‐like shape was vertically suspended (from the spine‐positioned mother) in this study, as in Yuryev et al. (2018), and a low‐melting temperature agarose was used to immobilize it. To assist agarose‐mediated immobilization of the uterus, we used (a) a polydimethylsiloxane (PDMS) membrane, which was cross‐incised at the center to narrowly pass (thus gently suspend/hold) the entire uterus above the mother (Figure 1d, 1); (b) an aspiration‐based transient holder (Figure 1d, 2), which was designed to gently pull (transiently, not continuously) the embryo's head through a small window made on the amniotic membrane and the uterine wall; (c) two metal bars that were designed to screw in from the opposite sides of a metal stage to hold the uterine sac containing the embryo to be observed (Y. Hattori et al., unpublished data; Figure 1d, 2); and (d) a coverslip that was stabilized onto the metal stage (Figure 1d, 3). Care was taken not to excessively pull umbilical vessels or compress the embryo during manual (finger‐mediated) embryo positioning (to ensure an “upright” posture with its head and the dorsal cerebrum at the top, opposite of the placenta, placing the head closer to the objective lens, Figure 1a).

#### 3.1.6 Duration and rate of successful observations

These procedures were easily applied to long uterine horns containing five or more fetuses. In short uterine horns containing only three to four fetuses, desired handling of the uterine sac and the fetus for imaging as described above was not possible. In 21 observation trials done in the present protocol using such “long” uterine horn cases, the duration of monitoring without unacceptable tilting of the field and also keeping the fetus healthy (with enough blood circulation) varied from 0 min (i.e., the field was already tilted and untrackable or the fetus was already unhealthy at 5 min) to 90 min (averaged duration was 20 min). “Successful” observations (25× monitoring with 5‐min intervals could be continued longer than 10 min, with at least three recording opportunities) were performed in seven cases (33%; averaged duration was 44 min). Three cases (14%) could be reliably monitored until 60 min (two H2B‐EGFP cases and one Fucci case), and these two 60‐min H2B‐EGFP cases were used for quantitative analysis of mitosis and IKNM. Besides the 21 cases, 40 more pilot trials including cases that used the “short” uterine tubes were performed with unsuccessful but technically valuable results.

#### 3.1.7 In utero observation of NPC dynamics near the apical surface of cerebral walls using an in toto cell‐labeling system

To monitor the mitosis of NPCs at the apical (ventricular) surface and IKNM of NPCs toward and away from the apical surface, we utilized transgenic mice in which all cell nuclei can be fluorescently visualized based on the localization of H2B protein (Kurotaki et al., 2007). Although labeling via in utero electroporation (IUE) enables monitoring of cerebral cortical NPC behaviors in slice culture (Konno et al., 2008; Okamoto et al., 2013) and intravital 2PM observation of IUE‐labeled embryos has become available for neurons in developing cerebral walls (Higuchi et al., 2016), we found that use of H2B‐EGFP mice was advantageous for more easily obtaining optical fields of interest (i.e., VZ regions abundantly containing dividing NPCs). When IUE‐labeled embryos were subjected to intravital 2PM, finding ideally labeled NPCs in the VZ was difficult, and excessive searching of appropriate fields damaged the embryos. Previous studies revealed that mitosis and IKNM in NPCs visualized in slices prepared from H2B‐mCherry mouse embryos (Okamoto et al., 2013; Shinoda et al., 2018) were equivalent to those observed during sporadic visualization of NPCs (Konno et al., 2008; Miyata et al., 2001; Okamoto et al., 2013). Technically, monitoring at 5 μm from the apical surface is useful for capturing (a) apical‐ward migration of a G2‐phase NPC's nucleus, (b) mitosis of that NPC, and (c) basal‐ward IKNM of G1‐phase daughter cells generated by that NPC (Okamoto et al., 2013; Shinoda et al., 2018). We therefore scanned the intrauterine cerebral walls of E13 H2B‐EGFP mice to find cells at 5 μm from the apical surface as a main target (Figure 2a).

Following the identification of a G2‐like cell's nucleus, a highly fluorescent metaphase plate was observed (condensed chromosome, observed at 0–5 min in Figure 2b) was then separated horizontally (observed at 10 min in Figure 2b with almost random planar orientation along the apical surface), which was further followed by the departure of a daughter cells’ nuclei to a more basal VZ region (by 45 min for cell a and by 55 min for cell b in Figure 2b). The sequential occurrence of these steps for apical cytogenesis, as well as the density of the total live H2B‐labeled cells, were seemingly indistinguishable from those observed in 3D cultures (Okamoto et al., 2013; Shinoda et al., 2018), but we sought to perform more quantitative comparisons, as follows.

### 3.2 Comparison of apical cytogenesis between slice culture and in utero systems

Since apical mitoses of NPCs are known to occur mostly horizontally (parallel to the apical surface; Hayder, Ang, & Rakic, 2003; Konno et al., 2008; Kosodo et al.., 2004; Landrieu & Goffinet, 1979; Smart, 1973), identifying separation of a metaphase plate into two chromosomal condensations, which can be completely captured at our focusing plane (5 μm from the apical surface), was chosen as the most reliable way for live counting of mitoses. We compared the frequency of occurrence of such identified mitosis events per unit time period (60 min) and per unit apical‐surface area (60 μm × 60 μm). As shown in Figure 3a, the frequency was 7.59 ± 1.14 in 3D culture (obtained from three separate fields of an E13 cerebral wall), reproducing the results obtained in previous studies that used E13 cerebral wall cultures (Okamoto et al., 2013; Shinoda et al., 2018). As shown in Figure 3b, the frequency of mitosis occurrence was 8.00 ± 1.00 in in utero 2PM (three separate fields from two independent E13 embryos), and no statistically significant difference was obtained between these two groups (*p* = .7, exact Wilcoxon rank‐sum test; Figure 3c).

Since it was still possible that mitoses under our 3D culture and mitoses under intravital 2PM are both similarly less frequent than they are in physiological situations, we further obtained the density of metaphase cells and that of metaphase plus anaphase cells from freshly fixed E13 in vivo specimens (not only from 3D culture and intravital 2PM, as shown above) and compared the metaphase densities between these three groups. Comparison revealed no statistically significant differences (*p* = .46 between 3D culture and intravital 2PM; *p* = .57 between 3D culture and fixed in vivo specimen; *p* = .79 between intravital 2PM and fixed in vivo specimen, *n* = six 60 μm × 60 μm fields in each group, exact Wilcoxon rank‐sum test; Figure 3d,e).

We next focused on the timing of departure of the daughter cells’ nuclei from the 5 μm level. Previous slice culture‐based studies showed that daughter cells generated by apically dividing NPCs move their nuclei away from the apical surface toward the basal side sequentially, with one daughter cell's nucleus/soma leaving more quickly (within 30–40 min after its “birth”, as technically defined by the separation of its mother cell's metaphase plate) than its sister cell's nucleus/soma (within 50–60 min; Okamoto et al., 2013; Okamoto et al., 2014; Shinoda et al., 2018). Slice culture has suggested that this sequential departure is due to each NPC's basal process being inherited solely by one of the two daughter cells and used for quicker nucleokinesis through it (Miyata et al., 2001; Saito et al., 2003), thereby avoiding a bottleneck problem in the periventricular space (Okamoto et al., 2013). Whether such sequential departure also occurs in vivo has not yet been addressed. As exemplified in Figure 2b, we observed that pair‐generated daughter cell nuclei sequentially disappeared from the 5 μm level (which was defined as their “departure”; *n* = 2 out of two cases in which departure of the nucleus was observed successfully in both daughter cells). Shorter monitoring (*n* = 15 cases) allowed us to capture only one daughter cell's nuclear departure, also suggesting that daughter cells’ nuclear departure was not simultaneous. Time from birth to departure (measured in such early‐departing daughter nuclei) was comparable between the 3D cultures (*n* = 13) and the intravital 2PM specimens (*n* = 17; *p* = .5563, exact Wilcoxon rank‐sum test; Figure 3f).

### 3.3 Fucci‐mediated detection of NPC dynamics in utero

**Figure 4:**
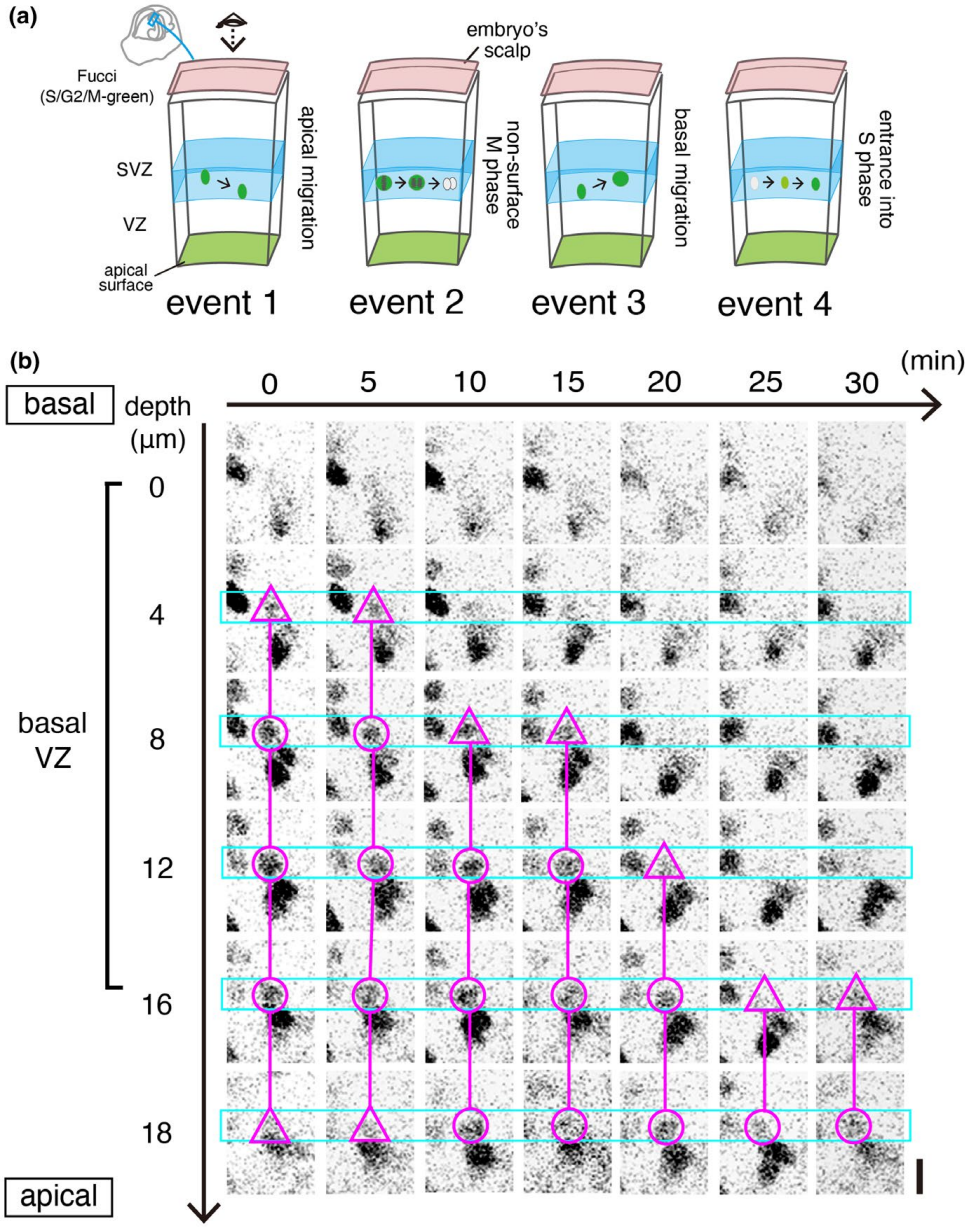
Intravital 2PM observation of Fucci (S/G_2_/M‐Green) mice. (a) Schematic illustration of four different cell‐cycle‐associated events that can be captured in the non‐surface zone (light blue, from a basal part of the VZ to the adjacent SVZ) of the cerebral wall using the Fucci (green) system in utero. Event 1, apical‐ward movement of a G2‐phase NPC's nucleus (an mAG^+^ nucleus moves). Event 2, progression of M phase (mAG disappearance at late M phase) in a non‐surface‐dividing NPC (whose soma is large and round‐shaped). Event 3, basal‐ward movement of the nucleus or soma of a G2‐ or M‐phase NPC, which is exhibited prior to non‐surface division (event 2). Event 4, entrance of an NPC into S phase (mAG expression begins in a relatively small nucleus). (b) A time series showing apical‐ward nuclear migration typical to G2‐phase NPCs (corresponding “event 1” of Figure 4a) in an E14 Fucci embryo in utero. Scanning was performed from the basal VZ (0 μm) to the middle VZ (18 μm). Initially (0 min), the tracked mAG^+^ nucleus was most clearly detected from 8 μm to 16 μm (magenta circle) with faint/peripheral fluorescence at 4 μm and 18 μm (magenta triangle). By 25 min, it moved apically and exited the basal VZ. Scale, 10 μm

For comprehensive assessment of the brain‐forming cell‐production dynamics, monitoring of cell‐cycle progression in NPCs with Fucci (S/G_2_/M‐Green) transgenic mice, in which cells in S, G2, and early M phases emit fluorescence of monomeric Azami Green (mAG) on the basis of the up‐regulation of human Geminin (hGem) (Sakaue‐Sawano et al., 2008; Watanabe et al., 2018), is useful. In a non‐surface NPC zone (from a basal part of the VZ to the adjacent SVZ), mAG fluorescence can be used for detecting four different cell‐cycle‐associated NPC events (Figure 4a). Firstly, after completion of S phase, G2‐phase NPCs’ nuclei start to migrate apically (Kosodo et al., 2011; “event 1” in Figure 4a). Secondly, since progression of M phase to its late‐stage results in sudden disappearance of an mAG^+^ soma‐like signal (Sakaue‐Sawano et al., 2008), extinction of mAG fluorescence in SVZ, which is the a second (non‐surface) place for mitosis (Haubensak, Attardo, Denk, & Huttner, 2004; Miyata et al., 2004; Noctor, Martinez‐Cerdeno, Ivic, & Kriegstein, 2004; Figure 1c), indicates that an NPC is successfully progressing into the late M phase (“event 2” in Figure 4a). Thirdly, since previous slice culture showed that non‐surface NPC mitoses are preceded by basal‐ward movement of G2‐phase nuclei or early M‐phase somata (Miyata et al., 2004), such migration (“event 3” in Figure 4a) is also expected to occur in utero. Finally, if a new mAG^+^ nucleus emerges in this zone, one likely scenario is that an NPC just after completion of G1 phase is entering S phase there (“event 4” in Figure 4a). An alternative scenario that the nucleus of an NPC that was already in S phase at a different position came to the observed position is also possible, although the latter is theoretically less likely, because a previous in vivo study showed that nuclear movement is minimal during S phase (Hayes & Nowakowski, 2000).

**Figure 5:**
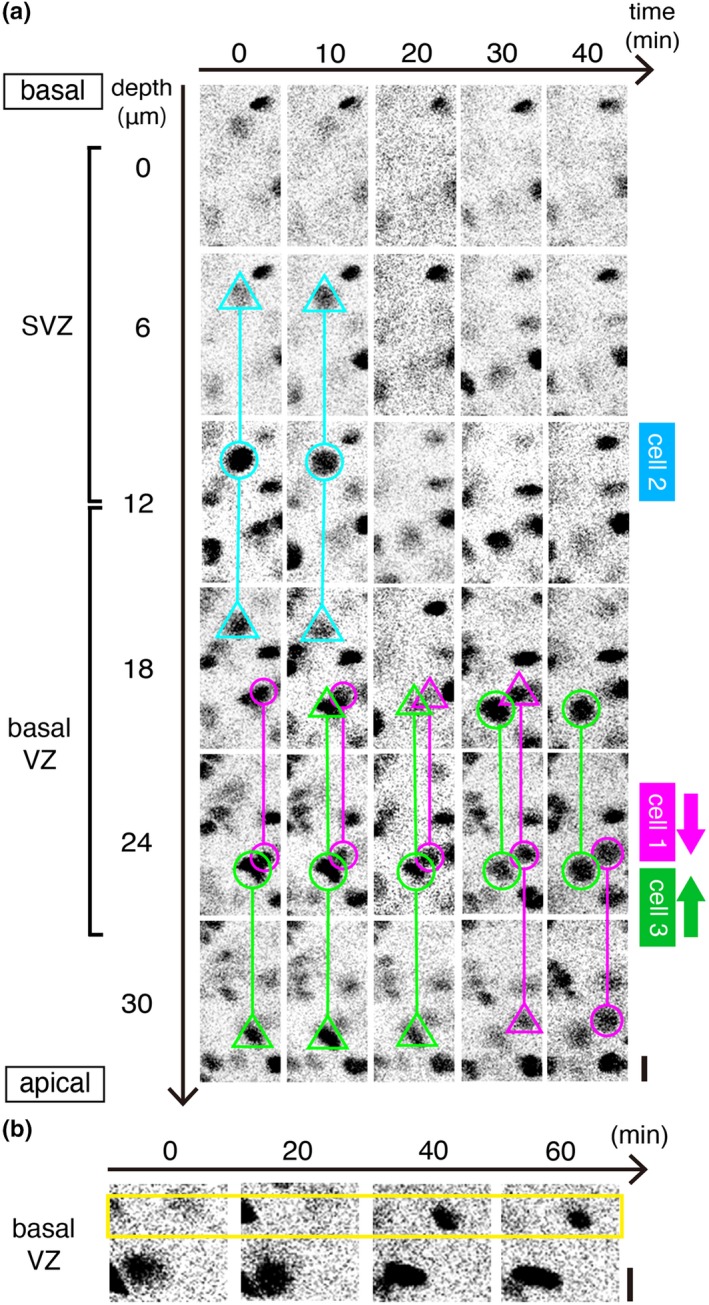
Intravital 2PM that captured cell‐cycle‐associated events including the extinction and the emergence of mAG signal in Fucci embryos in utero. (a) A time series showing cell‐cycle‐associated NPC dynamics in the SVZ and the basal VZ of an E14 Fucci (S/G_2_/M‐Green) embryo. Scanning was performed from the SVZ (0 μm) to the middle VZ (30 μm). Cell 1 (magenta) showed apical‐ward nuclear migration (corresponding to “event 1” of Figure 4a), as also shown in Figure 4b, typical to G2‐phase NPCs. Cell 2 (light blue) was initially brightly mAG^+^, large in size and round in shape, and its mAG then became undetectable, typical phenomenon in M‐phase‐progressing (dividing) cells (corresponding to “event 2” of Figure 4a). Cell 3 (green) exhibited basal‐ward movement (corresponding to “event 3” of Figure 4a), a phenomenon preparative for non‐surface mitosis. Scale, 10 μm. (b) A time series showing emergence of a new mAG^+^ nucleus in the basal VZ, indicative of either chronological entrance of a G1‐finishing NPC into S phase (corresponding to “event 4” of Figure 4a) or spatial entrance of an S‐phase NPC nucleus into the basal VZ. Scale, 10 μm

As shown in Figures 4b and 5, our horizontal sectional observation at a basal VZ region revealed that the density of mAG^+^ nuclei was lower than the density expected for total S‐phase nuclei that would have been labeled with bromodeoxyuridine in that depth (data not shown), suggesting a cell‐to‐cell variation in the expression of mAG‐hGem during S phase. These technical characteristics of our Fucci mice were disadvantageous to live counting the entire population of intra‐VZ NPCs that are entering, proceeding, or exiting S phase (i.e., obtaining frequency of initiation, progression, or calculating S‐phase nuclei per unit area and time, as is done for occurrence of apical mitoses using H2B‐EGFP mice [Figure 3b], was not possible). However, it was advantageous for clear observation of individual S‐ or G2‐phase NPCs that did not overlap with neighboring fluorescent nuclei.

Figure 4b shows that one mAG^+^ nucleus was moving apically at a velocity of 2 μm/min. This velocity was comparable to the maximal velocity observed for apical IKNM in slice culture (1.5–2.0 μm/min, while averaged velocity for apical‐ward IKNM in slice culture was 0.5 μm/min [Okamoto et al., 2014]). A similar apical IKNM case is shown also in Figure 5a (cell 1). In total, apical movement was observed in three mAG^+^ nuclei (two independent embryos). Figure 5a also shows that one soma‐like mAG signal (cell 2, round‐shaped and large) in SVZ suddenly disappeared, which was typical to division of NPCs in slice culture (Sakaue‐Sawano et al., 2008). In total, such non‐surface M‐phase progression was observed in two cells (one embryo). Our intravital 2PM also observed that a similarly large mAG^+^ object moved basally (Figure 5a, cell 3), suggesting that it was a G2‐phase nucleus or an M‐phase soma prior to non‐surface mitosis. We separately observed the emergence of a new mAG^+^ nucleus (smaller than cells 1–3) in the basal VZ (Figure 5b). It is possible that this new mAG^+^ cell entered S phase there. But, the present study cannot conclude so, because this Figure 5b case could not clearly be monitored over wider range (i.e., at different Z planes as done for Figures 4b and 5a), requiring more reliable distinction between the chronological entrance of an NPC into S phase here and the nuclear/somal movement of an S‐ or G2‐phase nucleus into this Z level.

## 4 DISCUSSION

Aiming at live observation of cell‐production dynamics by NPCs in the developing cerebral cortex in vivo, we tried to establish in utero 2PM using H2B‐EGFP or Fucci (S/G_2_/M‐Green) transgenic mouse fetuses growing. Compared to the previous similar intravital observation of developing cerebral cortices focused on superficial (outer) zones filed with differentiated neurons (Ang et al., 2003; Higuchi et al., 2016; Yanagida et al., 2012; Yuryev et al., 2016), our target (the VZ) was deeper (~300 μm deep from the coverslip at E13 and ~400 μm at E14). The use of a 2PM system with a GaAsP light detector was effective for sensitive signal acquisition at approximately 300 μm deep (though clear deeper observation was not possible). The observation depth achieved in this study is much smaller than that achieved in a recent 2PM that targeted the adult cerebral cortex (1 mm or even deeper; Kawakami et al., 2013). Multiple layering of different tissues (uterine wall, amniotic membrane, embryo's scalp and subcutaneous tissue, and brain wall), instead of a relatively more homogeneous adult cortical structure, seems to be an obstacle. In addition to this limitation in visibility along the *Z*‐axis, our pilot trials experienced that the deeper (>200 μm) zones were more easily tilted along the *X*–*Y* plane than they were at superficial (<100 μm) levels. This required the present study to carefully immobilize the fetus and the uterine sac. The efforts for effective immobilization, with sequential use of multiple holding devices (aspirator, metal bars, and agarose gel), gradually improved the visibility, and it was also effective to secure fetal circulation. Successful cases were, however, limited to 33% (as indicated by ≥10 min observations) and 14% (for 60 min observations; as shown in Figures 2, 3, 4) of the total trials (*n* = 21), even after establishing the present protocol. Although we cannot specifically identify what is insufficient and how it can be overcome at this point, our experiences establishing the present protocol suggest that improving the careful monitoring of placental and fetal circulation or better fetus‐holding procedures would be beneficial.

Despite the aforementioned overall difficulty in performing intravital 2PM monitoring of NPC dynamics, our quantitative assessment of cases that were judged to be healthy for at least 60 min (based on sufficient cranial blood flow and heart beat after imaging) revealed that NPC divisions at the apical surface of cerebral walls occurred at a normal frequency (with results similar to those from non‐2PM‐observed, completely physiological in vivo cerebral walls; Figure 3). Such NPC divisions occurring at a normal frequency were accompanied by IKNM behaviors to and from the apical surface, which were also similar to the results observed in 3D cultures (Figures 2, 4 and 5). This indistinguishability between NPC behaviors in 3D cultures and those in utero (as revealed in this study) is strong support for 3D culture to be used as a good method for studying cell‐production dynamics. On the other hand, 3D culture lacks the in vivo obstacles that fetuses must overcome (i.e., 3D‐cultured cells are “spoiled” with enriched conditions) and also has geometrical limitations.

In utero 2PM system to monitor the developing organs is necessary to capture real in vivo cellular behaviors or morphogenetic events that cannot be reproduced in 3D culture, although the present study could not reach such an ideal level due to insufficiency in observation length. Events to be studied by much improved in utero 2PM systems in the future would include long‐range or complicated cellular migration routes that are transected by slice preparations and possible interactions between brain‐forming cells and the surrounding cranium‐forming cells that are mostly lost during dissection and slicing steps. Entrance of blood vessels, whose involvement in brain‐cell production is suggested (Komabayashi‐Suzuki et al., 2019; Tan et al., 2016), and neural crest‐derived pericytes into brains (Yamanishi, Takahashi, Saga, & Osumi, 2012) would be interesting targets for in utero 2PM.

In addition, the in utero 2PM will provide a new platform to study the possible influences of risks in maternal–fetal relationships, such as acute ischemic influence on the fetus, on developing cells’ behaviors; it will facilitate studies in gynecological and neonatological viewpoints. For example, how NPCs are sensitive or resistant to transient or prolonged ischemia can be studied by intravital 2PM in combination with quantitative assessment of both apical and non‐surface mitoses and IKNM (via H2B‐EGFP and Fucci mAG) and the entrance into S phase (via Fucci mAG). Although this intravital 2PM study (at E13 or E14) was performed through windowing the uterine wall, we have shown that fetuses subjected to bona fide in utero observation keeping the uterine wall intact (at E15) could be healthily delivered (Y. Hattori et al., unpublished data). Further improvements would enable us to connect the intravital 2PM observation (under maternal perturbation) of NPCs in an in utero growing mouse (fetus) and the postnatal observation of that mouse (pup). Such analyses would widen organogenesis studies in developmental biology toward understanding systemic and whole‐life homeostasis.

## AUTHOR CONTRIBUTIONS

R.K. performed all imaging analyses and wrote the manuscript. T.S. supervised R.K. and wrote the manuscript. Y.H., Ka.S., and Ke.S. contributed to 2PM observation and assisted in data analysis. T.Q.P., Y.T., and A.S. contributed to aspiration devices. S.K. and T.K. assisted in maternal care and evaluation of fetal circulation. M.N. and T.F. assisted in the use of the H2B‐EGFP mice and the 2PM systems. T.M. designed the project and wrote the manuscript.
