# Supplementary figures and images for: Two‐photon microscopic observation of cell‐production dynamics in the developing mammalian neocortex in utero
Source: Dev Growth Differ. 2020 Jan 14;62(2):118–28. doi: 10.1111/dgd.12648 (PMC7027555; doi:10.1111/dgd.12648)

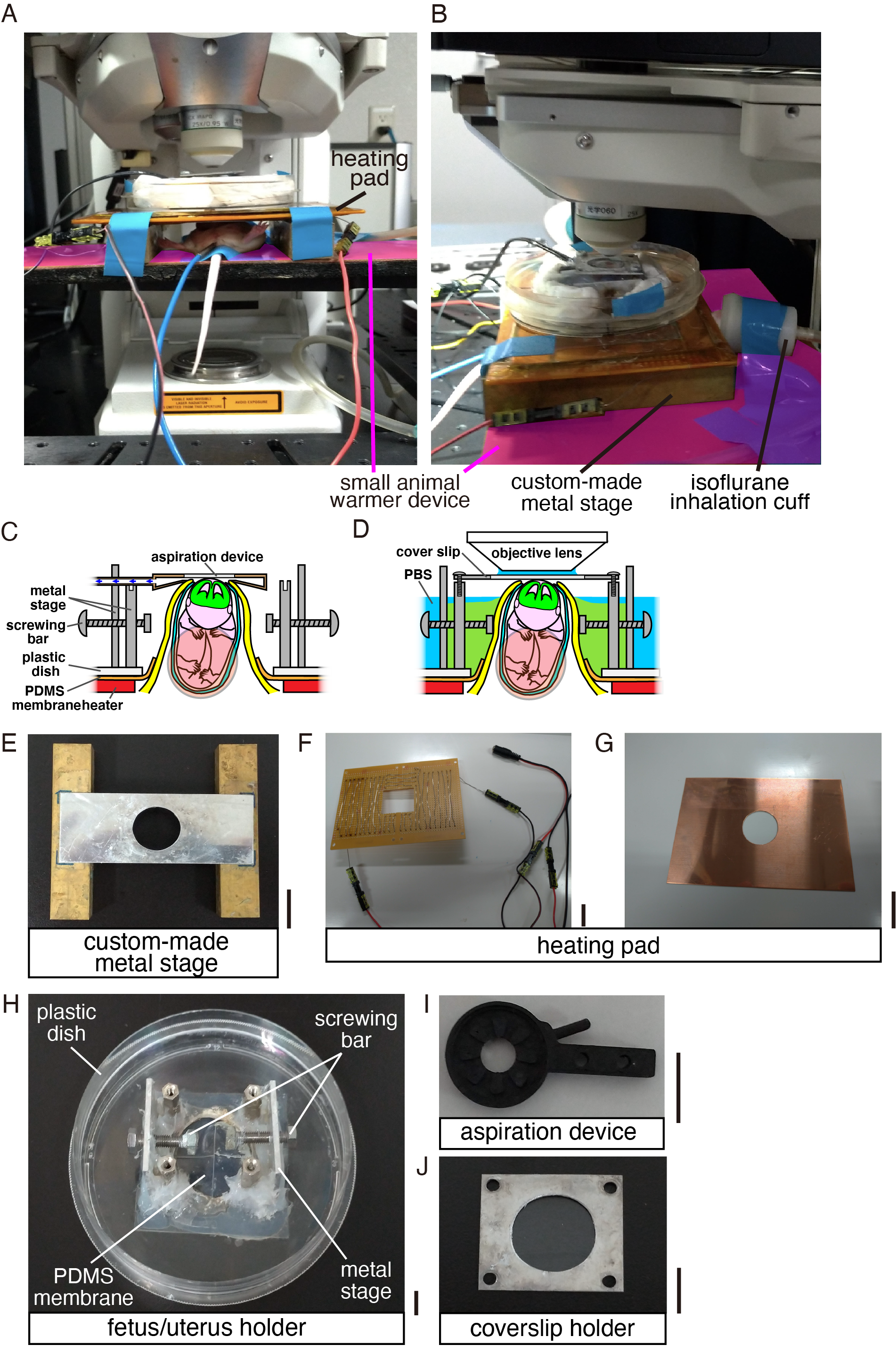

Supplement: Supplementary file 1 [file DGD-62-118-s001.tif]
