# Supplementary material for: Two‐photon microscopic observation of cell‐production dynamics in the developing mammalian neocortex in utero
Source: Dev Growth Differ. 2020 Jan 14;62(2):118–28. doi: 10.1111/dgd.12648 (PMC7027555; doi:10.1111/dgd.12648)
Supplement: Supplementary file 5 [file DGD-62-118-s005.docx]

**Supplemental Fig. 1.**

Custom-made devices for *in utero* 2PM observation of NPCs in the cerebral wall of E13–14 mouse embryos.

(A, posterior view) and (B, side view) Pictures showing how a pregnant mother mouse was positioned on a warming device on the microscopic stage following anesthetization with isoflurane; the image also shows how the uterus (pulled out from the abdominal cavity of the mother) was immobilized and warmed, and how the fetus was coverslipped. (C) and (D) Sectional schematic illustrations of the device system used to immobilize the fetus and the surrounding uterine sac (corresponding to Fig. 1D). (E, top view). Custom-made metal stage on which the uterus-immobilizing device (H) was placed. Its height was set to be just above the belly of the mother mouse. (F and G, top views) Heating pad (for the uterus/fetus) consisting of a nichrome wired circuit board (F) and a copper plate (G). (H, top view) Uterus-immobilizing device composed of (1) 10 cm-diameter polystyrene dish whose center was apertured (2.5 cm diameter) and covered with a PDMS membrane and (2) a metal stage equipped with two screwing bars. (I, top view) Aspiration device to hold the embryo’s head gently and transiently (J, top view). Holder for a glass coverslip. Scale, 2 cm in E, F and G; 1 cm in H, I and J.

**Supplemental Movie 1**

Intravital 2PM of an E13 H2B-EGFP mouse, scanning from the scalp through the cerebral wall to reach the lateral ventricle (at >200 μm deep). Corresponding to Fig. 2A

**Supplemental Movie 2**

Intravital 2PM (in an E13 H2B-EGFP mouse) that captured division of an NPC near the ventricular surface and departure of two daughter cells’ nuclei. Corresponding to Fig. 2B

**Supplemental Movie 3**

Intravital 2PM (in an E13 H2B-EGFP mouse) that captured periventricular division of several NPCs in a 60 μm x 60 μm field. Corresponding to Fig. 3B.
